# Efficient Use of Graphene Oxide and Silica Fume in Cement-Based Composites

**DOI:** 10.3390/ma14216541

**Published:** 2021-10-30

**Authors:** Ahmad Abdullah, Mohamed Taha, Mohamed Rashwan, Mohamed Fahmy

**Affiliations:** 1 Civil Engineering Department, College of Engineering, University of Bisha, Bisha 61922, Saudi Arabia; 2 Civil Engineering Department, Faculty of Engineering, Aswan University, Aswan 81542, Egypt; 3 Civil Engineering Department, Faculty of Engineering, Assiut University, Assiut 71515, Egypt; Muhammedhtaha@gmail.com (M.T.); mohamed.rashwan@aun.edu.eg (M.R.); 4 School of Energy Resources, Environmental, Chemical and Petrochemical Engineering (EECE), Egypt-Japan University of Science and Technology (E-JUST), Alexandria 21934, Egypt

**Keywords:** cement composites, graphene oxide, silica fume, mix design

## Abstract

Incorporation of graphene oxide (GO) and silica fume (SF) to cement composites enhances their mechanical properties if suitable proportional amounts of GO and SF are used. This study presents a simplified approach to determine experimentally the optimum GO and SF contents that should be added to the cementitious mixture to obtain a proper and stable dispersion of GO sheets within the cement matrix. Composite mortar specimens with different GO and SF contents were designed and tested under flexural and compression loading. The phase formation and the microstructure of selected samples were also investigated to give an in-depth interpretation of the test results. The main criterion to determine the GO and SF contents was the ultimate strength required of the GO–cement composite. It was found that there was a composite interaction between the SF and GO contents in the cementitious mixture, which an envelope surface could describe if all other mix design parameters are kept constant.

## 1. Introduction

Recently, carbon nanomaterials have been widely used to enhance the mechanical properties of cementitious composites [1,2,3,4,5,6,7]. They can also refine the composites’ microstructure to obstruct the ingression of aggressive materials [8,9,10,11] or improve electrical conductivity [12,13]. A minute percentage of not more than 0.1% of such nanomaterials could diminish the innate defects of cement composite of brittleness, porosity, and low conductivity. The basic structural unit of all allotropes of these carbon nanomaterials is graphene [14]. It is a 2D single layer of carbon atomic crystal arranged in a hexagonal lattice. The carbon atoms are connected in between by a very strong σ-bond and a *π*-bond. The σ-bond is responsible for the exceptional mechanical properties of the graphene sheet, with a tensile strength of 130 GPa and Young’s modulus close to 1 TPa. At the same time, the weak *π*-bond makes the graphene sheet available for chemical modification. Commonly, to reduce the hydrophobicity attribute of graphene, it is modified with oxygen-containing functional groups, producing graphene oxide (GO) [15].

As with all carbon nanomaterials, the effectiveness of GO in enhancing the mechanical and rheological properties of cement composite is highly affected by the quality of its dispersion in the cement matrix [16,17]. Although the oxidization process slightly lowers the mechanical properties of the GO sheet, it is considered a first critical step in achieving well-dispersed graphene in cement pore solution [18]. Moreover, the functional groups could physically help to adsorb the metal ions released from the cement hydration process and form strong bonds with the C-S-H products [19]. Therefore, the produced cement composite could benefit from the excellent mechanical properties of the 2D GO sheet. For instance, introducing a mild percentage of 0.05% GO by weight of cement could increase both the compressive strength and the flexural strength of the cement paste (CP) by up to 33% and 59%, respectively [20]. The addition of GO to CP increases none vaporable water and the calcium hydroxide contents, which enhances the degree of hydration in the cement paste, producing more C-S-H products [21]. Moreover, the GO sheets could effectively diminish crack propagation by restricting sub-microcracks from transforming into microcracks and providing high cracking resistivity [22].

The second step to improve the GO performance in the cement composite is to overcome the possible re-agglomeration of graphene sheets in the cement matrix. One of the main reasons that would trigger such aggregation is the formation of a very basic medium after the dissolution of cement minerals: alite (C_3_S), belite (C_2_S), aluminate (C_3_A), and ferrite (C_4_AF), which increases the concentration of the divalent calcium ions (Ca^++^) within the cement matrix at the early stages of the hydration process [23,24]. Many measures have been used before to maintain the dispersion of the GO within the cement matrix, such as mechanical ultra-sonication and the use of surfactants [25,26]. The former could be effective in dispersing the GO in aqueous solutions before incorporation into the cement matrix. However, an extra energy dose of sonication could harm the morphology of graphene sheets [27]. The latter, however, could be more effective to nicely disperse the GO in cement matrix if a suitable surfactant is used and premixed with cement slurries. The improper surfactant may reduce the bond characteristics of the GO sheets with the cement matrix, increase the amount of entrapped air, or delay the hydration reaction of cement [28,29,30].

The silica fume (SF) has been widely used to produce high-performance cement composites. It physically contributes as a nanofiller to densify the cement matrix, which is positively reflected on both the microstructure and mechanical properties. It also chemically reacts with portlandite (CH), in what is known as the pozzolanic reaction, producing more C-S-H products [31,32]. Some recent studies have claimed that this reaction could be considered responsible for reducing Ca++ concentration at the early stages of the hydration process, resulting in improved dispersion of GO within the cement matrix [12,33,34]. However, others have discredited this argument, reporting the possible unstable behavior of SF-GO in cement paste solution (CPS) by the formation of larger size SF-GO aggregation [35,36]. This controversy prompts the need to have a study to investigate the possibility of the existence of interaction between GO and SF at different concentrations within the cement matrix and to determine its limits that could improve their dispersion and stability in cementitious composites.

To serve this purpose, 20 cement composite mortar mixes were designed with different GO and SF contents. The composite specimens were tested under flexural and compression loading at the ages of 7 and 28 days to be compared with benchmark samples. Moreover, the composite specimens’ phase formation and microstructure were selectively investigated, using X-ray diffraction (XRD) and scanning electron microscopy (SEM), respectively, to explain how the GO and SF could enhance the mechanical performance of the composite. Finally, a simplified approach was introduced for the efficient design of cementitious composites. The method relates the accumulative specific surface area (SSA) of GO and SF, as a physical property, to mechanical strength enhancement. Therefore, an optimum ratio of GO/SF could be determined. 

## 2. Materials and Methods

### 2.1. Materials

**Figure 1:**
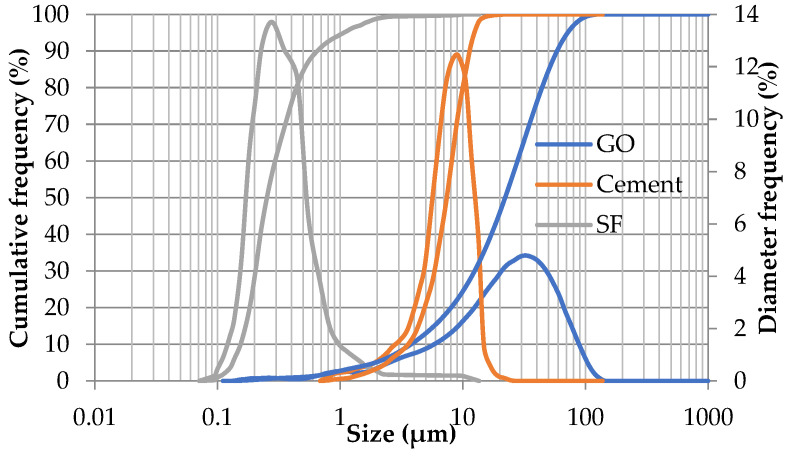
Particle size distribution of cement, SF, and GO sheets.

A 42.5 N ordinary Portland cement (OPC) conforming to the requirements of European Standard Specifications BS EN 1-197/2011 [37] and SF, supplied by KIMA Co. Ltd. (Shanghai, China) for chemical industries, were used in this study. The chemical compositions of both cement and SF are shown in Table 1. The particle size distributions of cement and SF, measured by a laser particle size analyzer BT-2001 (Dandong, China), are also shown in Figure 1. The average SSA of the cement and SF samples were 0.424 m^2^/gm and 23.138 m^2^/gm, respectively. The SSA of GO determined using the low-temperature adsorption of nitrogen (BET method) was 2.973 m^2^/gm, which is much lower than the theoretical value (≈2418 m^2^/gm) [38]. Similar values have been reported before in the literature [39,40,41]. The used sand was standard sand with silica content not less than 90% passing from standard sieve No. 10 (2 mm) and totally retained on standard sieve No. 200 (74 μm), according to BS EN 196-1 [42]. The average SSA of sand, determined based on the average logarithmic diameter method, was 0.673 m^2^/gm.

**Figure 2:**
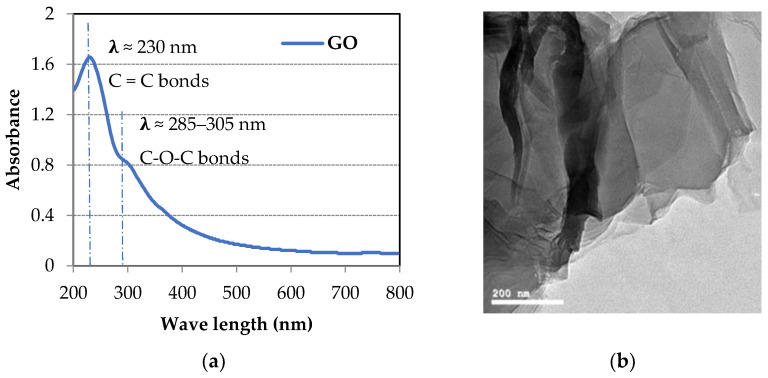
(**a**) UV–vis spectrum; (**b**) TEM image of GO sheets.

The GO was prepared using Hummer’s method [43] in Nanotech Co. Ltd. for Photo Electronics (Cairo, Egypt). The provided GO was dispersed in water with 5 mg/mL concentration, 3–5 stacked layers, and PH of 7. The size distribution of the GO sheets was also checked using the laser particle size analyzer and then normalized to the particle size distribution of the cement and SF, as shown in Figure 1. The Ultraviolet-visible (UV–vis) spectrum of the GO dispersion, along with a transmission electron microscopy (TEM) image, is shown in Figure 2.

### 2.2. Specimen Preparation

In this study, a total number of 20 mixes of cement composite mortar were prepared according to ASTM C109/C109-16a [44]. Each mix has nine prisms with a dimension of 40 × 40 × 160 mm^3^. All specimens were cast with a filler to binder (cement and SF) ratio of 2.75:1 and a water-to-cement ratio of 0.485. Different percentages of GO were used—namely, 0.03%, 0.05%, 0.07%, and 0.09% of the total weight of binder materials. In 15 mixes out of 20, the SF partially replaced the cement by 5%, 7%, and 9% of the weight. The mix proportions are shown in Table 2.

In specimens in which the SF was used, the SF was first mixed with diluted GO suspension and stirred for 2 min. Then, cement was added into the GO–SF mixture and mixed for an additional 4 min using an automatic mixer with designated speeds and a fixed mixing sequence. Next, the composite mortar was placed in the casting molds and covered with a non-permeable flat surface. After 24 hours, the specimens were removed from molds and then put in water until testing. It should be mentioned that the workability of the composite mixture gradually diminished, while the amounts of both GO and SF gradually increased, and the water-to-cement ratio was kept constant. The maximum reduction in the workability index, measured using the flow table test, was about 16% in specimens with high incorporated ratios of GO and SF such as S9G5, S9G7, and S9G9.

### 2.3. Specimen Testing

To evaluate the GO/SF possible interaction on enhancing the mechanical behavior of cement composite, flexural and compression tests were carried out at the ages of 7 and 28 days after curing, according to ASTM C348-14 [45]. The machine used for mechanical testing was a MEGA 10-1000-50 DM1-S (FORM+TEST, Riedlingen, Germany) with a maximum capacity of 50 KN for compression and 10 KN for bending, and a bending roller length of 100 mm. The load was automatically increased through digital controller DIGIMAXX^®^ C-20 (FORM+TEST, Riedlingen, Germany) at the designated rates. The loading rates for the flexural and compression tests were 0.05 kN/S and 2.4 kN/S, respectively. According to ASTM C348-14, a compression test is carried out using portions of prisms broken in flexural just after flexural test to determine the compressive strength. 

To investigate the microstructure of the hardened cement composite, the fracture surface of the crushed chunks from the strength tests were prepared and then examined by QUANTA FEG 250 Field Emission Microscope (FEI, Hillsboro, OR, USA). The SEM imaging was conducted on specific samples representing the low, medium, and high values of accumulative SSA. Samples were prepared on a monocrystalline silicon substrate and coated with a thin layer (5 nm) of gold. Furthermore, XRD was performed on dry ground samples, using the DIANO 2100E X-ray diffractometer (DIANO, Woburn, MA, USA) with Cu Kα (λ = 0.15424 nm), to identify the phase formation progression of the selected samples at 7 and 28 days.

## 3. Results

### 3.1. Flexural Strength of GO/SF Cement Composite

**Figure 3:**
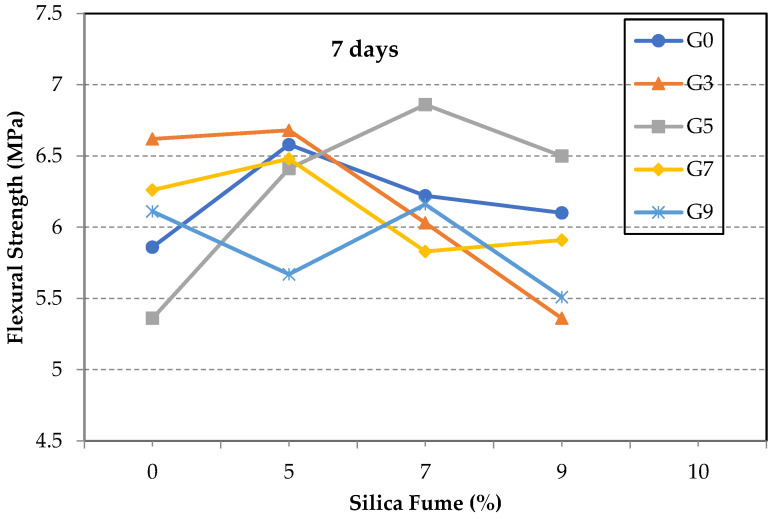
Flexural strength of test specimens at seven days.

Figure 3 shows the flexural strength of the GO/SF cement composite specimens at seven days. The specimens are grouped according to the GO content into series G0, G3, G5, G7, and G9. In most series with low GO ratios, the general trend is that the increase in SF percentage increases the tensile strength enhancement up to a certain level. After that, any increase in the amount of SF would result in an adverse effect. For instance, the maximum enhancement achieved at seven days was 17% for specimen S7G5, whereas the maximum enhancement achieved was 13% and 12% in specimens S0G3 and S5G0 when the GO or SF was individually incorporated in the mixture, respectively. However, the early strength gain of a composite containing a high GO ratio of 0.07% and 0.09% could not be significantly improved with the SF addition, as shown in Figure 3. These results clearly imply the existence of a positive composite action between the GO and SF up to a definite limit, after which a negative post-peak behavior occurred.

Different possible reasons may work individually or together, resulting in such negative post-peak behavior: the use of a high amount of SF that may prevent the interactions between GO sheets and hydration products by mechanically separating the GO sheets from the hydration products [33,34]; the high surface area of the resulting mixture, which probably consumes the mixing water, reducing the workability and hence hindering the completion of cement hydration reactions; the possible re-agglomeration of the GO sheets in basic mediums, especially when a high GO content is used. Other studies have come to the same results before [13,22,34].

**Figure 4:**
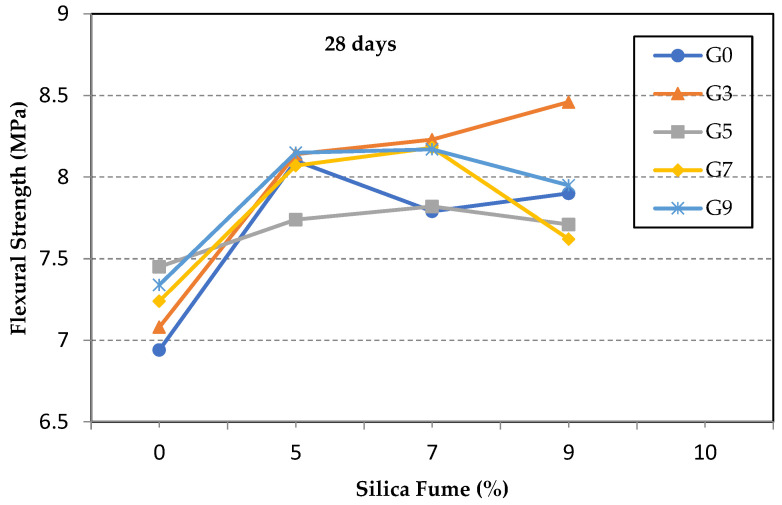
Flexural strength of test specimens at 28 days.

The 28-day flexural strength tests are shown in Figure 4. It is observed that the percentage of strength gained for specimens with high GO ratios was lower than that at seven days. For instance, the improvement in the flexural strength of specimen S7G5 was about 13%, compared with 17% at seven days. This implies the role of GO in accelerating the hydration process at early stages, resulting in more dense and interlocked hydration products [23,46]. Another observed result reveals that those specimens with high SF content, compared with the GO ratio, started to gain more strength at 28 days than at 7 days. For instance, the percentage increase in the flexural strength for specimen S7G3 was 18%, compared with 3% at seven days. This is because the high SF content would delay the strength gained at early ages, as it is known that the pozzolanic reaction continues for later ages [32]. 

### 3.2. Compressive Strength of GO/SF Cement Composite

**Figure 5:**
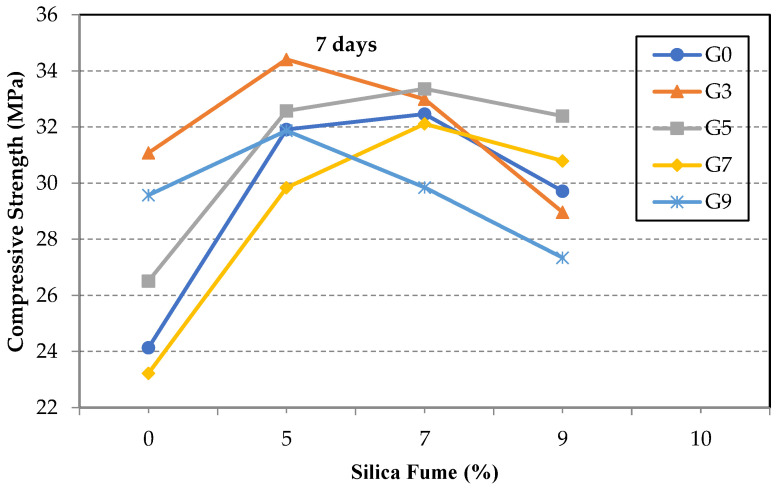
Compressive strength of test specimens at seven days.

The compressive strengths of the GO/SF cement composite specimens at seven days are shown in Figure 5. The results show nearly similar behavior to that of the flexural strength. In the absence of SF, the highest increase in the compressive strength may be achieved when a small amount of the GO is used to ensure proper dispersion of the GO sheets in the cement matrix. The increase in the compressive strength of specimen S0G3 was 29%. On the other hand, the incorporation of the SF was able to improve the performance of GO sheets in the composite; the SF densified the mixture and maintained the dispersion of the GO sheets. The increase in the compressive strength, for example, of specimens S5G3 and S7G5 were 43% and 38%, respectively. However, the increase in the compressive strength in their counterpart specimens S0G3 and S0G5, without SF, was only 29% and 10%, respectively.

**Figure 6:**
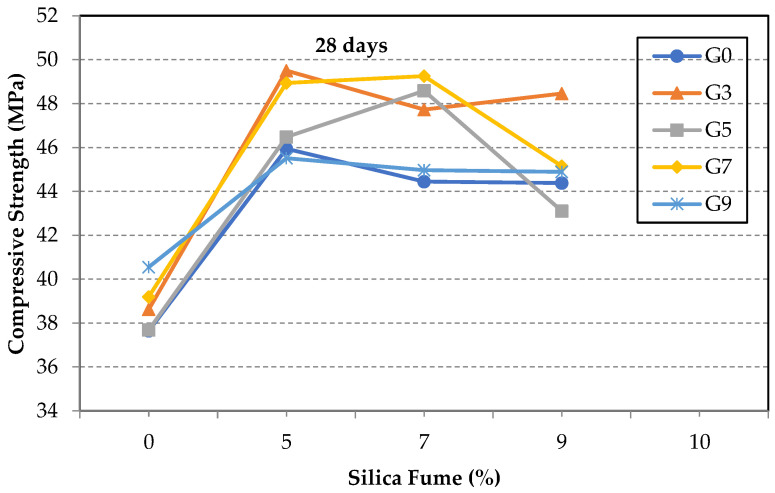
Compressive strength of test specimens at 28 days.

Figure 6 shows the compressive strength of the 28-day for the GO/SF cement composite. The maximum increase in the compressive strength was in specimens S5G3 and S7G5 by 31% and 29%, respectively. Notably, by inspecting the test results, it is clear that there is a peak point at which a proper amount of SF should be used to achieve the optimum strength at a given GO content in each group. Such amount of SF differs as the GO percentage varies but does not necessarily yield the same amount of strength improvement at a certain water/cement ratio. The same concept is still valid if a certain amount of SF is used in the mixture, while the GO percentage is the one that changes. Such finding guides the possibility of relating the strength enhancement in the composite to a physical property, which could be the nominal SSA of the incorporated additives. This is discussed in the following section. 

### 3.3. Microstructure of GO/SF Cement Composite

**Figure 7:**
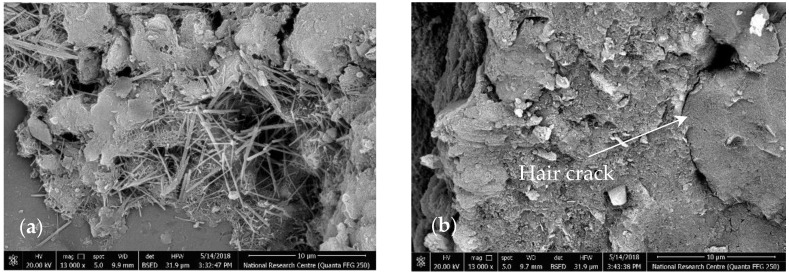

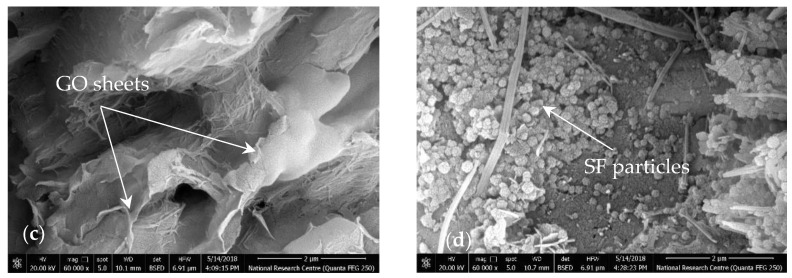
SEM images of the fracture surface at the age of 28 days: (**a**) sample S0G0; (**b**) sample S5G0; (**c**) sample S5G3; (**d**) sample S5G9.

The SEM imaging for the fracture surface of the tested specimens after 28 days of curing could clearly explain the strength results. Selective samples that represent different values for SSA are presented in Figure 7. For the reference specimen S0G0, Figure 7 shows massive growths of rod-like crystals (Ettringite) filling cracks. Such hair cracks usually congregate under the effect of loading in major cracks, resulting in a brittle failure.

On the other hand, the addition of SF to the mixture densified the cement matrix, as shown in specimen S5G0, Figure 7b. A pozzolanic reaction occurred between SF and the free lime (CH), producing additional CSH in many of the voids around hydrated cement particles. This additional CSH provided the mortar with improved strength, but it could not arrest the crack once it was developed. 

Figure 7c shows the SEM images of the fracture surface of specimen S5G3, where the addition of proportional amounts of GO and SF resulted in a denser microstructure. The image shows GO sheets covered with C-S-H products. The ettringite could still be seen inside the matrix but with a much smaller ratio, reducing its negative effect on developing weak points inside the cement matrix. The incorporation of GO stimulated the hydration process. The high surface area and the ratio of edge atoms increase the chemical reactivity of the GO sheet. The oxygen-containing functional groups, available on the GO sheet, offer a seeding effect to the cement hydrates and form strong bonds with the cement matrix, reducing the probability of sliding under the effect of loads [19,47]. Moreover, the GO sheet’s excellent mechanical properties help it bridge cracks perfectly [8] and offer an additional toughening mechanism. This was consistent with the previous mechanical testing results. Such strength enhancement would not be achieved without proper dispersion of both SF and GO sheets in the cement matrix.

With increasing the SF or GO amount in the mixture as in sample S5G9, the SSA of the additives reached a high value, resulting in a blockage and preventing the completion of the pozzolanic reaction. Unhydrated particles of SF could be easily identified on the magnified fracture surface of sample S5G9, as shown in Figure 7d. This explains the lower strength achieved of such samples. 

### 3.4. XRD Analysis of GO/SF Cement Composite

**Figure 8:**
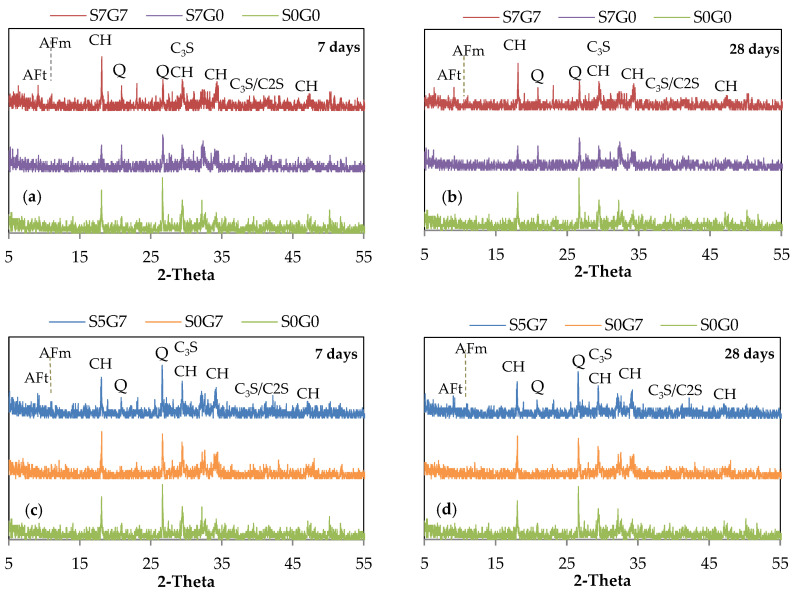
XRD analysis of GO/SF Cement composite: (**a**) GO effect at 7 days; (**b**) GO effect at 28 days; (**c**) SF effect at 7 days; (**d**) SF effect at 28 days.

The XRD spectrums of the GO/SF cement composite at the age of 7 and 28 days are shown in Figure 8. The main crystalline phases were portlandite (CH), ettringite (Aft), monosulfate (AFm), and unhydrated alite (C3S) and belite (C2S). The quartz (SiO_2_) phase was also present due to the mixture containing sand despite the grinding and sieving of the samples. Figure 8a presents the effect of incorporating the GO into a cement mixture containing SF (S7G0). By comparing the main portlandite peaks in specimen S7G0 with the reference specimen S0G0, no large differences could be observed for the peak intensities (100) and (101) crystal faces at the age of seven days. However, a lower main peak of the crystal face (001) could be observed for specimen S7G0. These results imply that the SF consumed the portlandite [48]. 

As the GO was added, a prominent peak (001) could be seen in specimen S7G7, which implies that the GO could promote the formation of the portlandite. This provides an opportunity for the SF to combine with portlandite, producing more C-S-H and hence a dense microstructure. The peak intensity of the portlandite lowered as time passed. These results are consistent with previous findings in the literature [23,28,49].

As shown in Figure 8b, the addition of GO to a plain mixture (S0G7) improved the hydration degree at early ages, which is evinced by the increased intensity of the main peak of portlandite (001), compared with specimen S0G0. The incorporation of SF to the cement composite (S5G7) was expected to lower the main peak of portlandite (001). However, no difference could be observed between the main peaks of samples S5G7 and S0G7 at different ages. This might be due to the addition of a small amount of SF (5%) and a higher amount of GO (0.07%). GO may offset portlandite consumption as a result of SF addition. 

### 3.5. CH Orientation at the Fracture Surface of GO/SF Cement Composite

The XRD pattern could determine the effect of incorporating GO and SF on the orientation of the CH at the fracture surface, where the orientation index could indicate the compactness of the hardened composite [30,48,50]. Generally, the lower the orientation index was, the more compacted matrix was achieved. In this study, the orientation index is calculated according to the following Equation [51]:

R = 1.35 × I_(001)_/I_(101)_ (1)

where I_(001)_ and I_(101)_ are the peak intensities of the (001) and (101) crystal planes, and the (101) crystal plane is used as a reference plane. 

**Figure 9:**
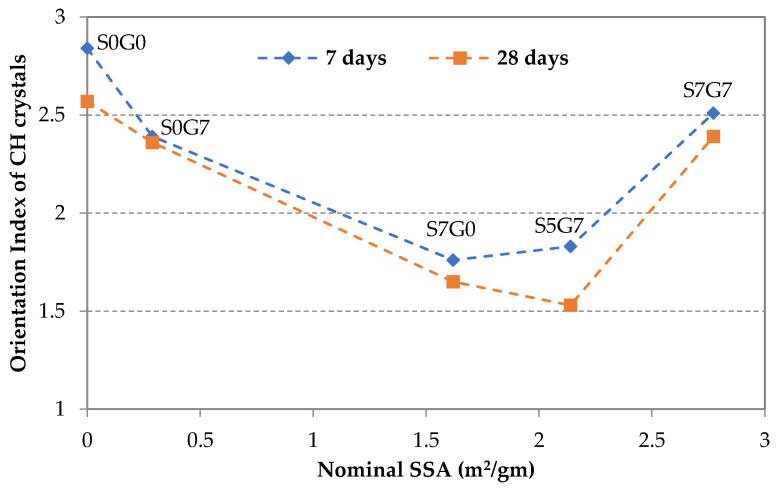
Orientation index of CH crystals.

The orientation index was calculated for the selected samples and plotted against the nominal sum of SSA_n_ for GO and SF, as shown in Figure 9. At the age of seven days, as shown in Figure 9, the orientation indexes for the composite samples were 2.39, 1.76, 1.83, and 2.51 for specimens S0G7, S7G0, S5G7, and S7G7, correspondingly, which were lower than 2.84 for the reference specimen. The values were much lower at 28 days; the lowest orientation index was nearly 1.53 for specimen S5G7. These results imply that the addition of GO and SF significantly lowered the orientation index of CH crystals, which indirectly enhanced the mechanical properties of the composite. 

Another observation indicates that the orientation index continued to decrease as the SSA increased up to a specific limit, after which the orientation index started to rise again. This behavior indicates the existence of an optimum SSA at which a dense matrix could be achieved, and hence a maximum strength enhancement. These results conform to the strength results mentioned above.

## 4. Estimation of Strength Enhancement in GO/SF Cement Composite

**Figure 10:**
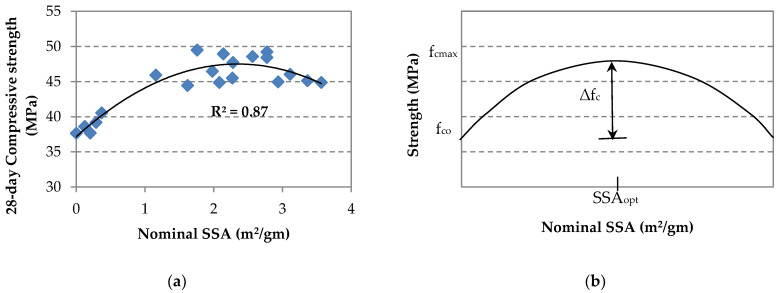
Strength enhancement to nominal SSA relationship: (**a**) compressive strength; (**b**) hypothetical strength behavior.

The results above clearly imply that the enhancement in the strength of the cement composite could be related to the nominal SSA of the incorporated GO and SF materials. Figure 10a shows the behavior of the composite strength (represented by the 28-day compressive strength) against the nominal sum of the SSA_n_ for the GO and SF. In this study, the SSA_n_ was determined as follows:

SSA_n_ = SSA_SF_ + SSA_GO_ (2)

The nominal SSA of GO (SSA_GO_) was determined according to the following equation [38]:

SSA_GO_ ≈ η [ SSA_G_ × 12/(12 × 0.737 + 16 × 0.263)] (3)

where SSA_G_ is the theoretical SSA of a single graphene sheet (≈2630 m^2^/gm) [52], 12 and 16 are the relative atomic weights of carbon and oxygen, respectively, based on the assumed formula of monolayer GO; C_0.737_O_0.263_H_x_ and η is an efficiency factor accounts for the dispersion quality of GO sheets in the mixture. This factor was calibrated against the experimental results using nonlinear regression and is given as follows:

η = [1 − 0.83 exp (−a SSA_SF_/b SSA_GO_)], b ≠ 0 (4)

where a and b are the percentages of the SF and GO in the mixture, respectively. This was justified based on the role of the SF in promoting the GO contribution in strength enhancement of the cement composite, as shown from the test results. Similar results have been reported before in the literature [12,13,35]. 

The strength enhancement could be described by an envelope surface, where the enhancement reaches the plateau at an optimum SSA_opt_, as depicted in Figure 10b. The maximum design strength (f_cmax_) is calculated as follows:

f_cmax_ = f_co_ + ∆f_c_ (5)

where f_co_ is the characteristic strength of the plain mixture, and ∆f_c_ is the enhancement of GO and SF additives. 

The optimum SSA_opt_ to produce a maximum strength enhancement ∆f_c_ of the additives could be evaluated as follows:

SSA_opt_ = (∆f_c_/k)^0.5^ (6)

where k is a constant that could be evaluated experimentally, using two-trial mixes incorporating two different percentages of GO and SF, along with the reference specimen S0G0 (represented by f_co_ on the curve). Having plotted the curve, both the optimum SSA_opt_ and the maximum strength enhancement ∆f_c_ could be evaluated from the curve.

This approach could be easily followed for a practical engineer to determine the optimum ratios of GO and SF to design a composite mix that exploits the constituent materials’ properties. Firstly, a particular amount of SF could be chosen, and the optimum amount of GO could then be determined. 

## 5. Conclusions

This study investigated the effective use of GO and SF as nanoadditives to cementitious material. In total, 20 composite mortar mixes were prepared and tested under flexural and compression loading, and the fracture surfaces were selectively investigated using SEM and XRD analysis. The results show a positive interaction between both the GO and SF that can enhance the strength of the composite better than adding any of them individually. For instance, when the GO and SF were incorporated into the cement matrix by certain proportional amounts such as 0.03% GO and 5% SF, the compressive strength enhancement could reach up to 43% at seven days. However, the strength enhancements were only 29% and 32% for GO and SF, respectively, if they were incorporated individually. The optimum strength enhancement could be achieved when certain amounts of both GO and SF were added to the cement matrix. At the same time, all other parameters, such as water/binder and binder/filler ratios, were constant. This composite interaction was adversely affected if either the GO or SF percentage increased. Finally, a simplified approach relating the strength enhancement to the nominal SSA of the additives (GO and SF) was presented to determine their optimum ratios.

## Figures and Tables

**Table 1:** Chemical properties of cement and SF.

Material	SiO_2_	Al_2_O_3_	Fe_2_O_3_	CaO	MgO	SO_3_	Na_2_O	K_2_O
Cement	23.77	5.41	6.2	57.14	2.28	2.25	0.64	0.25
SF	93.4	0.8	1.28	0.32	0.75	-	-	0.35

**Table 2:** Mix proportion of test specimen.

Test Specimen	Water/Cement Ratio	Binder:Sand	SF/Cement Weight %	GO/Cement Weight %
S0G0	0.485	1:2.75	0	0
S0G3	0.485	1:2.75	0	0.03
S0G5	0.485	1:2.75	0	0.05
S0G7	0.485	1:2.75	0	0.07
S0G9	0.485	1:2.75	0	0.09
S5G0	0.485	1:2.75	5	0
S5G3	0.485	1:2.75	5	0.03
S5G5	0.485	1:2.75	5	0.05
S5G7	0.485	1:2.75	5	0.07
S5G9	0.485	1:2.75	5	0.09
S7G0	0.485	1:2.75	7	0
S7G3	0.485	1:2.75	7	0.03
S7G5	0.485	1:2.75	7	0.05
S7G7	0.485	1:2.75	7	0.07
S7G9	0.485	1:2.75	7	0.09
S9G0	0.485	1:2.75	9	0
S9G3	0.485	1:2.75	9	0.03
S9G5	0.485	1:2.75	9	0.05
S9G7	0.485	1:2.75	9	0.07
S9G9	0.485	1:2.75	9	0.09

## Data Availability

Not applicable.
